# Ventricular-Subventricular Zone Contact by Glioblastoma is Not Associated with Molecular Signatures in Bulk Tumor Data

**DOI:** 10.1038/s41598-018-37734-w

**Published:** 2019-02-12

**Authors:** Akshitkumar M. Mistry, David J. Wooten, L. Taylor Davis, Bret C. Mobley, Vito Quaranta, Rebecca A. Ihrie

**Affiliations:** 1 Department of Neurological Surgery, Vanderbilt University Medical Center, Nashville, TN USA; 2 Vanderbilt-Ingram Cancer Center, Vanderbilt University Medical Center, Nashville, TN USA; 3 Department of Biochemistry, Vanderbilt University School of Medicine, Nashville, TN USA; 4 Department of Radiology, Vanderbilt University Medical Center, Nashville, TN USA; 5 Department of Pathology, Microbiology and Immunology, Vanderbilt University Medical Center, Nashville, TN USA; 6 Department of Cell and Developmental Biology, Vanderbilt University School of Medicine, Nashville, TN USA

## Abstract

Whether patients with glioblastoma that contacts the ventricular-subventricular zone stem cell niche (VSVZ + GBM) have a distinct survival profile from VSVZ − GBM patients independent of other known predictors or molecular profiles is unclear. Using multivariate Cox analysis to adjust survival for widely-accepted predictors, hazard ratios (HRs) for overall (OS) and progression free (PFS) survival between VSVZ + GBM and VSVZ − GBM patients were calculated in 170 single-institution patients and 254 patients included in both The Cancer Genome (TCGA) and Imaging (TCIA) atlases. An adjusted, multivariable analysis revealed that VSVZ contact was independently associated with decreased survival in both datasets. TCGA molecular data analyses revealed that VSVZ contact by GBM was independent of mutational, DNA methylation, gene expression, and protein expression signatures in the bulk tumor. Therefore, while survival of GBM patients is independently stratified by VSVZ contact, with VSVZ + GBM patients displaying a poor prognosis, the VSVZ + GBMs do not possess a distinct molecular signature at the bulk sample level. Focused examination of the interplay between the VSVZ microenvironment and subsets of GBM cells proximal to this region is warranted.

## Introduction

Neural stem cell niches are hypothesized to support malignant gliomas. The two widely recognized neural stem cell niches are the ventricular-subventricular zone (VSVZ)^1^ and the subgranular zone (SGZ)^2^. The former is located in the lateral linings of the lateral ventricles, and the latter is located in the dentate gyrus of the hippocampus^3,4^. While no correlation between patient outcome and glioblastoma (GBM) involvement with the SGZ is evident^5^, prior observations suggest that VSVZ contact by GBMs negatively impacts patient survival^5,6^.

These observations have rapidly led to at least 12 clinical studies assessing the benefit of incorporating VSVZ radiation in the standard GBM therapy regimen^7,8^. Eight of these studies did not show any benefit, and the benefit was minimal in the remaining 4. This lack of a therapeutic effect of VSVZ radiation is currently unexplained. Efforts to understand its basis must first address two gaps in our knowledge. First, a critical evaluation of the survival effect of VSVZ contact should ideally be adjusted for widely-recognized prognosticators of GBM patient survival, including extent of resection, tumor volume, and molecular features such as IDH mutation status, glioma CpG island methylator phenotype (G-CIMP), and *MGMT* promoter methylation status^5,6,9^. Second, whether GBMs with VSVZ contact or involvement (here termed VSVZ + GBMs) are molecularly different from VSVZ − GBMs is unknown. To date, there is little evidence to indicate whether VSVZ + GBMs are enriched for a specific molecular subclass or other genomic signature relative to VSVZ − GBMs^10–17^.

To address this critical gap in our understanding of the clinical and molecular differences in VSVZ + GBMs and VSVZ − GBMs, the association between patient survival and glioblastoma contact with the VSVZ was rigorously and comprehensively tested in two independent patient datasets. Further, computational analyses were conducted on the molecular data available in the TCGA to identify any evident molecular signatures of VSVZ + GBMs and/or VSVZ − GBMs.

## Methods

### Patient Datasets and Clinical Data Collection

Approval from an ethical standards committee (the Institutional Review Board) to conduct this study was received (Study IRB# 161891). Patient consent was waived. 170 consecutive adult (>18 years of age) patients who received a cytoreductive, maximal safe resection of a supratentorial GBM between 2011 and 2017 were identified (33% overlap with prior data^5^). Following resection, all patients were considered for treatment with radiation and temozolomide according to the Stupp protocol^18^. Their clinical course was followed up to July 2017. Their age, preoperative Karnofsky performance status score (KPS), whether they received postoperative radiotherapy and temozolomide and completed the Stupp protocol regimen, their GBM molecular status (i.e., *MGMT* promoter methylation and *IDH1/2* mutation), and overall (OS) and progression free (PFS) survivals were collected. Another 254 patients from The Cancer Imaging Archive (TCIA)^19^ with contrasted brain imaging and corresponding OS data in The Cancer Genome Atlas (TCGA-GBM) database were identified (Table S1)^20^. Their demographic, clinical, and molecular information was obtained from the TCGA-GBM database^20^. Detailed treatment information on these patients was retrieved from Level 1 clinical data available from the Broad Institute’s Genome Data Analysis Centers Firehose (http://firebrowse.org/). Patients who received adjuvant radiation of at least 60 Gy and completed at least 6 cycles of adjuvant temozolomide were noted. All data generated or analyzed during this study are included in this published article (and its Supplementary Information files) and are publicly available from the TCIA/TCGA databases. An analyzed institutional clinical dataset is available from the corresponding author on reasonable request.

### Radiographic Data Collection

**Figure 1:**
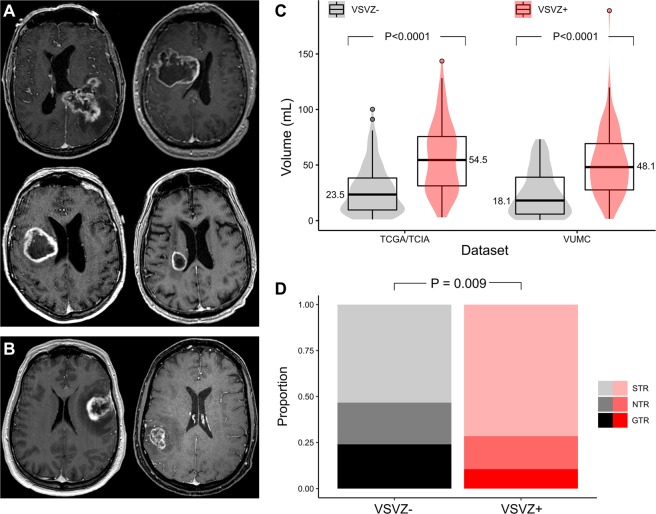
Pre- and post-operative radiographic characterization of glioblastomas. Examples of pre-operative MRI images from the TCGA/TCIA-GBM dataset depicting glioblastomas with ventricular-subventricular zone contact ((**A**), 4 VSVZ + GBMs) and without VSVZ contact ((**B**), 2 VSVZ − GBMs). Distributions and boxplots of the tumor volumes based on VSVZ contact status of glioblastomas in the TCGA/TCIA and institutional (VUMC) datasets are depicted in (**B**). Median volumes (written next to the boxplot) of VSVZ + GBMs were significantly greater than volumes of VSVZ − GBMs in both datasets. The distributions of extent of resection (subtotal, near total, and gross total; STR, NTR, and GTR, respectively) based on VSVZ contact status assessed on post-operative MRIs in the institutional dataset is shown in (**D**). Extent of resection was greater in VSVZ − GBMs.

Magnetic resonance images of the brain were available for all patients, except for 3 in the TCIA/TCGA-GBM dataset who had computed tomography images. The initial preoperative post-contrast brain imaging of all patients was assessed for VSVZ contact of GBM independently by two reviewers (neurosurgeon and a board-certified neuroradiologist) without knowledge of patient outcome. Using OsiriX Lite software (version 9.4, Pixmeo, Geneva, Switzerland), VSVZ + GBMs were identified by the contact or involvement of the post-contrast tumor enhancement with the lateral ventricular ependyma^5,6^, the location of the VSVZ in human brain^4^. Agreement was 86%. Disagreements were resolved by a consensus radiological review. Figure 1 depicts representative images of VSVZ + GBMs (Fig. 1A) and VSVZ − GBMs (Fig. 1B) from the TCGA/TCIA-GBM dataset. GBM contact with the ependyma of the third (n = 5 and n = 6 in the institutional and TCGA/TCIA-GBM datasets, respectively) or fourth ventricles (n = 2 and n = 0, respectively) was not considered when determining VSVZ contact status. Tumor volume was calculated using the semiautomated volume rendering function in OsiriX Lite after delineating the outer edge of a tumor’s contrast enhancement^5^. In cases of multifocal tumors, their volumes were summed. For all the above assessments, axial imaging sequences were used; when clarification was needed, they were reformatted into coronal or sagittal sequences. In the institutional dataset, extent of resection was assessed independently by a neurosurgeon and neuroradiologist using postoperative, post-contrast magnetic resonance imaging obtained within 24 hours after the operation. Gross total (GTR) and subtotal resections (STR) were judged respectively by the absence or presence of residual tumor contrast enhancement. A portion of the STRs were deemed near-total resections (NTRs) if there was less than 5% residual tumor or if the neuroradiologist could not definitely exclude minuscule residual. The time at which the first postoperative, radiographic evidence of tumor recurrence or progression (PFS) agreed upon by both a neuroradiologist and neuro-oncologist (i.e., ruling out radiation necrosis or pseudoprogression) was also noted. PFS data were missing in 68 patients (26.8%) in the TCIA/TCGA-GBM dataset. Because two-thirds of these patients (23, 67.6%) died within the 90-day postoperative period (defined as the postoperative global period by the U.S. Centers for Medicare & Medicaid Services^21^), they were considered as disease progression for purposes of PFS analysis.

### Molecular Data Collection

Molecular data of the 254 TCIA/TCGA-GBM patients (Table S2) were retrieved from either the GBM dataset (data version 2016_01_28) in the Broad Institute’s Genome Data Analysis Centers Firehose (http://firebrowse.org/) or the GBM provisional cohort in the cBioPortal for Cancer Genomics (http://cbioportal.org)^22,23^. These data included gene mutations and copy number alterations (from cBioPortal) as well as DNA methylation, gene expression (mRNA sequence and microarray performed on the three available platforms—Agilent 244 K Custom Gene Expression, Affymetrix HT Human Genome U133, and Affymetrix Human Exon 1.0 ST Array), and reverse phase protein array (from Broad Institute). No re-processing or re-normalization was performed on the data.

### Data Analysis

#### General Statistical Analysis

Standard descriptive statistical methods are used to report variables and compare distributions of continuous and proportions of categorical variables. Statistical significance α was set at ≤0.05 (two-sided) for all statistical analyses. In instances of multiple simultaneous hypothesis testing, *P* value was adjusted to judge its significance and control false-discovery rate using the Benjamini–Hochberg procedure^24^. The Benjamini–Hochberg critical value for a false discovery rate was set at 5%. R version 3.4 (R Foundation for Statistical Computing, Vienna, Austria) together with its publicly available packages were used for all analyses.

#### Survival Analysis

Survival time points were censored at the latest time of follow-up if patients were alive or their living status was unknown (for OS) or at the last time of intracranial imaging if they had no evidence of tumor progression/recurrence (PFS). Both univariate log-rank (Mantel–Cox) and multivariate Cox proportional hazards regression survival analyses were performed to test the impact of VSVZ contact on survival, and the data are reported as hazard ratios (HRs) with 95% confidence intervals (CIs). Proportional hazards assumption in the multivariate Cox analyses was tested by assessing the significance of the relationship between Schoenfeld residuals and time for the overall model. If this assumption was not met, a time-dependent covariate in the model was incorporated as a piecewise function with regards to time to confirm reproducibility of the results^25^. Survminer R package (Version 0.4.0) was used to perform these analyses, calculate median survival times, and plot right-censored Kaplan-Meier curves.

#### Molecular Data Analyses

Differences in gene mutations and copy number alterations between VSVZ + GBMs and VSVZ − GBMs were explored using Fisher’s exact test. Differences in gene methylation, gene expression, and protein expression between VSVZ + GBMs and VSVZ − GBMs were assessed using the Mann-Whitney U test due to their global non-normal distributions.

Further computational analyses conducted included: a weighted gene co-expression network analysis^26^; partial least squares followed by logistic regression^27^ to detect linear combinations of gene methylations, gene expressions, and protein expressions predictive of VSVZ + GBMs and VSVZ − GBMs; nonlinear dimensionality reduction of the multi-dimensional gene methylation, gene expression, and protein expression datasets using t-SNE^28^ to segregate VSVZ + GBMs and VSVZ − GBMs in high-dimensional space; and unsupervised consensus clustering to reveal whether gene and protein expression clustered based on VSVZ contact^29^. Methods for these analyses are detailed in Supplemental Methods. Of the several gene expression datasets, Affymetrix HT Human Genome U133 was used to represent results. Results of these analyses on other datasets are available upon request.

## Results

### VSVZ Contact Independently Stratifies Survival

**Figure 2:**
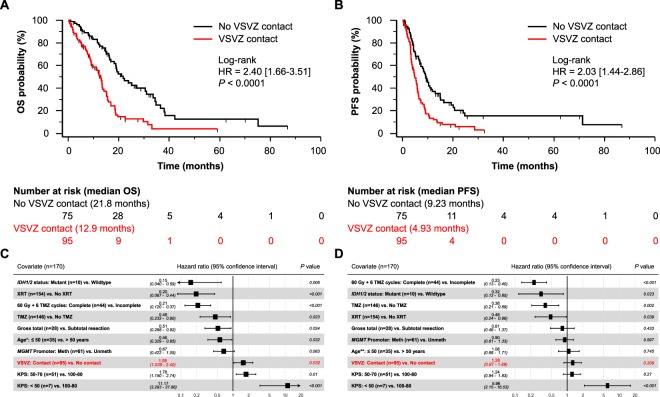
Patients with ventricular-subventricular zone-contacting glioblastoma (VSVZ + GBM) have decreased survival in the institutional dataset. Kaplan-Meier survival curves depict overall survival (OS, **A**) and progression free survival (PFS, **B**). Censored values, representing alive patients or unknown living status, are indicated with tick marks underneath the survival curves. (**C**,**D**) Forest plots of hazard ratios (HRs) obtained from multivariate Cox overall and progression free survival analyses. *HRs with 95% confidence intervals for age when treated continuously is 1.02 [1.00–1.04], *P* = 0.03, for OS and **1.00 [0.98–1.01], *P* = 0.83, for PFS; significance of VSVZ contact remained unchanged.

In the VUMC institutional dataset (n = 170), median OS of patients with VSVZ + GBMs (n = 95, 55.9%) was significantly lower compared to those with VSVZ − GBM (12.9 vs. 21.8 months; log-rank HR 2.40 [1.66–3.51], *P* < 0.0001; Fig. 2A). Patients with VSVZ + GBM also had a lower PFS (4.93 vs. 9.23 months; log-rank HR 2.03 [1.44–2.86], *P* < 0.0001; Fig. 2B). Extent of resection classified as GTR (100% resection), NTR (>95%), and STR (<95%) was, respectively, 10, 17, 68 in VSVZ + GBMs and 18, 17, 40 in VSVZ − GBMs (*P* = 0.009 Mann-Whitney U test for difference; Fig. 1D).

Adjusting for the known confounders including extent of resection (treated as an achievement of GTR or not) in a multivariate Cox survival analysis, VSVZ contact remained an independent predictor of OS (HR 1.58 [1.04–2.40], *P* = 0.03; Fig. 2C) but not PFS (HR 1.28 [0.87–1.89], *P* = 0.21; Fig. 2D). Univariate survival analyses of the confounders are presented in Table S3. Treating extent of resection as a ternary variable [i.e. GTR (100% resection), NTR (>95%), and STR (<95%)] did not significantly change the effect size or the significance of VSVZ contact in OS or PFS; NTR was not a predictor of OS (HR 0.71 [0.41–1.21], *P* = 0.21) or PFS (HR 0.72 [0.44–1.17], *P* = 0.19). Surgical resection for recurrence was performed on 17 VSVZ + GBM and 10 VSVZ − GBM patients (*P* = 0.53).

**Figure 3:**
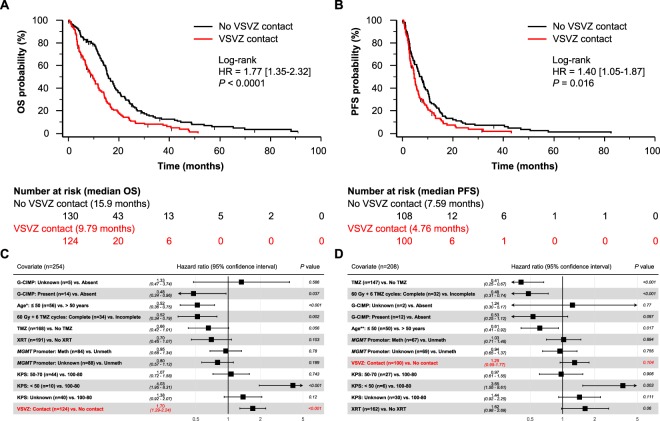
Patients with ventricular-subventricular zone-contacting glioblastoma (VSVZ + GBM) have decreased survival in the TCIA/TCGA-GBM dataset. Kaplan-Meier survival curves depict overall survival (OS, **A**) and progression free survival (PFS, **B**). Censored values, representing alive patients or unknown living status, are indicated with tick marks underneath the survival curves. (**C**,**D**) Depict forest plots of hazard ratios (HRs) obtained from multivariate Cox overall and progression free survival analyses. *HRs with 95% confidence intervals for age when treated continuously is 1.03 [1.02–1.04], *P* < 0.001, for OS and **1.02 [1.00–1.03], *P* = 0.009, for PFS; significance of VSVZ contact remained unchanged.

VSVZ contact by GBM was also noted to be an independent predictor in the TCIA/TCGA-GBM dataset. Compared to the institutional dataset, this dataset included proportionally fewer patients who received temozolomide and radiation therapy and more patients with *MGMT* promoter methylated GBMs (Table 1). 124 (48.8%) of the 254 total patients were classified as VSVZ + GBM. Median OS of these patients was significantly lower compared to those with VSVZ − GBM (9.79 vs. 15.9 months; log-rank HR 1.77 [1.35–2.32], *P* < 0.0001; Fig. 3A). Patients with VSVZ + GBM also had a lower PFS (4.76 vs. 7.59 months; log-rank HR 1.40 [1.05–1.87], *P* = 0.016; Fig. 3B). Adjusting for the available confounders in a multivariate Cox survival analysis, VSVZ contact remained a significant predictor of OS (HR 1.70 [1.29–2.24], *P* < 0.001; Fig. 3C), but not PFS (HR 1.29 [0.95–1.77], *P* = 0.1; Fig. 3D). Because IDH mutation status was unavailable in 51 (20%) and 41 (19.7%) patients in the OS and PFS TCIA/TCGA-GBM datasets, respectively, its surrogate G-CIMP status (unavailable in 5 (2%) and 2 (1%) patients, respectively) was used to adjust the effect of VSVZ contact status.

**Table 1:** Demographics and clinical characteristics of the two cohorts analyzed in the study.

Covariate	TCIA/TCGA-GBM cohort (n = 254)	VUMC Cohort (n = 170)	*P* value*
Age, median [IQR]	59.5 [52, 69]	61.0 [51.8, 68.6]	0.79*
Age ≤ 50, n (%)	56 (22.0%)	35 (20.6%)	0.81
KPS, median [IQR]	80 [60–80]	80 [70–90]	0.86*
100–80	160/214 (74.8%)	112 (65.9%)	0.07
70–50	44/214 (20.6%)	51 (30.0%)	0.04
Under 50	10/214 (4.7%)	7 (4.1%)	1
Unknown	40/254 (15.7%)	—	—
Temozolomide, n (%)	168 (66.1%)	146 (85.9%)	<0.0001
Radiation Therapy, n (%)	191 (75.2%)	154 (90.6%)	<0.0001
IDH Mutants, n/total (%)	12/203 (5.9%)	10 (5.9%)	1
IDH Status Missing, n/total (%)	51/254 (20.1%)	—	—
G-CIMP Status, n/total (%)	14/249 (5.6%)	—	—
MGMT Promoter Methylation, n/total (%)	84/166 (50.6%)	60 (35.3%)	0.006
MGMT Promoter Methylation Status Missing, n/total (%)	88/254 (35.6%)	—	—
Extent of Resection			
Gross total	—	28 (16.5%)	—
Near total	—	34 (20.0%)	—
Subtotal	—	108 (63.5%)	—
Tumor Volume (cm^3^), median [IQR]	33.0 [16.1–60.4]	36.2 [16.9–58.6]	0.66*
VSVZ Contact, n (%)	124 (48.8%)	95 (55.9%)	0.17

Abbreviations: IQR – interquartile range, KPS – Karnofsky Performance Scale score, VSVZ – ventricular-subventricular zone; *P* values marked with an * asterisk are obtained from Mann-Whitney U tests, all others are obtained from Fisher’s Exact tests.

**Figure 4:**
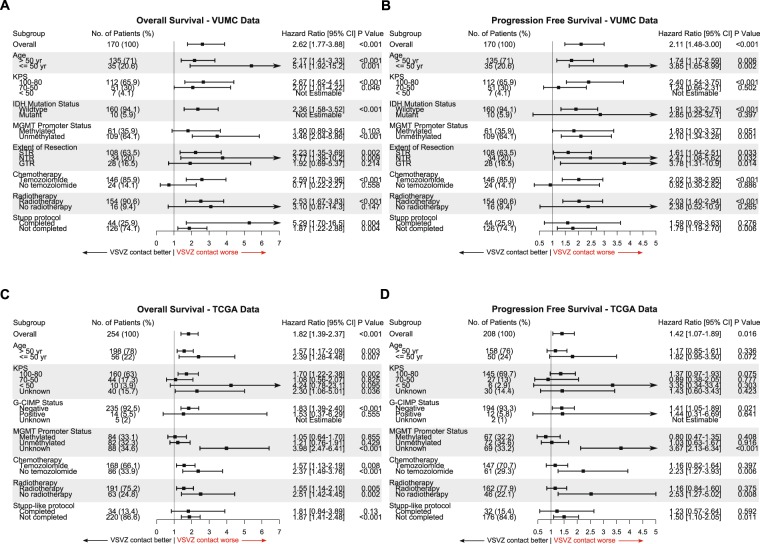
Ventricular-subventricular zone-contacting glioblastomas (VSVZ + GBMs) associate with decreased survival in a majority of the subgroups of patients. Forest plots of hazard ratios associated with VSVZ + GBM obtained from subgroup univariate Cox survival analyses in the institutional (**A**,**B**) and TCGA/TCIA-GBM **(C**,**D**) datasets are plotted. (**A**,**C**) Represent overall and (**B**,**D**) represented progression free survival subgroup analysis. Hazard ratios are plotted on the x-axis with 95% confidence intervals (CIs).

Confounder-based subgroup survival analyses revealed that VSVZ + GBMs were associated with decreased overall and progression free survival in the majority of the subgroups (Fig. 4). Interestingly, in both datasets, the effect of VSVZ contact on survival was greater in younger patients 50 or less years of age. This effect was maintained in an adjusted survival analysis. Patients ≤50 years of age in TCIA/TCGA-GBM and institutional datasets had higher multivariate HRs for overall survival (1.69 [0.80–3.58], *P* = 0.17, and 4.04 [1.29–12.6], *P* = 0.016, respectively) compared to patients >50 years of age (1.46 [1.08–1.99], *P* = 0.02, and 1.52 [0.96–2.42], *P* = 0.075). This effect was especially clear with exclusion of patients with secondary (*IDH1/2* mutant) GBMs or G-CIMP positive GBMs, which are known to have a higher survival profile and incidence in younger patients (TCIA/TCGA-GBM dataset: HR 3.33 [1.36–8.16], *P* = 0.008, 11/56 (20%) patients excluded; institutional dataset: HR 3.79 [1.21–11.8], *P* = 0.02, 5/35 (14.3%) patients excluded).

### VSVZ + GBMs Do Not Display Distinct Molecular Signatures

**Figure 5:**
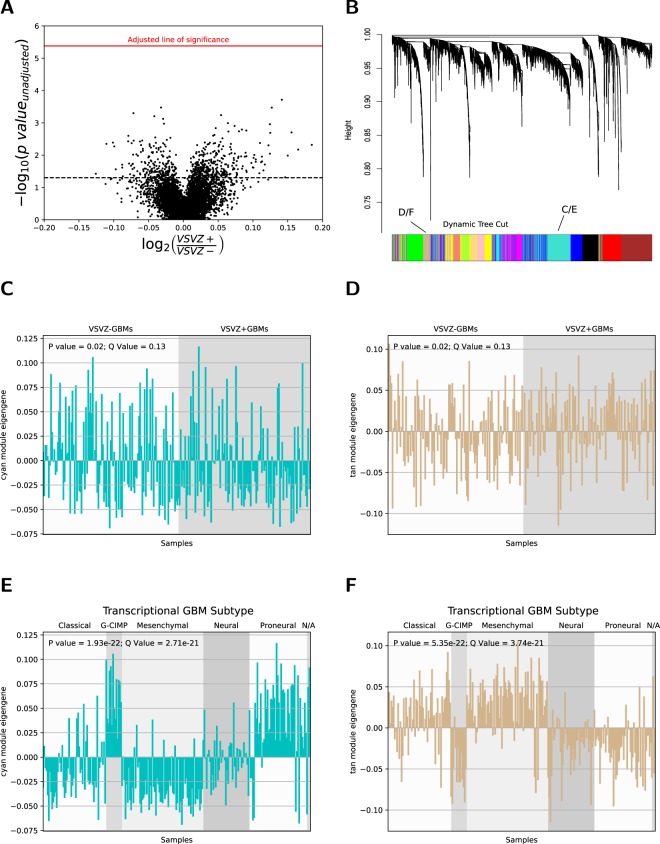
Gene expression does not identify ventricular-subventricular zone-contacting glioblastomas (VSVZ + GBMs) in the TCIA/TCGA samples. (**A**) Volcano plot of differential gene expression between VSVZ + GBMs and VSVZ − GBMs. Log_10_ of the unadjusted p-value of genes (dots) is plotted against fold change (the log_2_ of the ratio of mean gene expression in VSVZ + GBMs and VSVZ − GBMs). Dotted black line represents unadjusted (*P* = 0.05) and red line represents adjusted (*P* = 4.15 × 10^−6^) threshold of significance. (**B**) Dendrogram and the results of its dynamic cuts obtained from weighted gene co-expression network analysis of the Affymetrix HT Human Genome U133 gene expression dataset from TCGA. Fourteen modules of coexpressed and coregulated genes defined by the dynamic tree cuts are represented with blocks of color. Module eigengene (expression variation of genes within the module; i.e, high module eigengene expression denotes high expression of genes within that module) of the cyan (**C**) and tan (**D**) modules, the two examples depicted, was assessed in each sample and compared between VSVZ + GBMs and VSVZ − GBMs. There is no significant difference in cyan and tan module eigengenes between VSVZ − GBMs and VSVZ + GBMs; however, a significant difference in cyan and tan module eigengenes based on the transcriptional subtype was noted (**D** and **E**).

Given their divergent survival profiles, molecular markers from the 254 TCIA/TCGA-GBM patients were analyzed to identify possible differences between VSVZ + GBMs and VSVZ − GBMs. No single gene mutation (Table S4), copy number variation (Table S5), gene expression (Fig. 5A, Table S6), protein expression (Table S7), or DNA segment methylation (Table S8) met the statistical threshold to be confidently deemed as different between VSVZ + GBMs and VSVZ − GBMs groups with the sample size used. Re-analysis pre-filtering 50% of the genes with minimal global variation, which decreases the penalty imposed by multiple hypothesis adjustment to increase discoveries^30^, did not alter these results.

**Figure 6:**
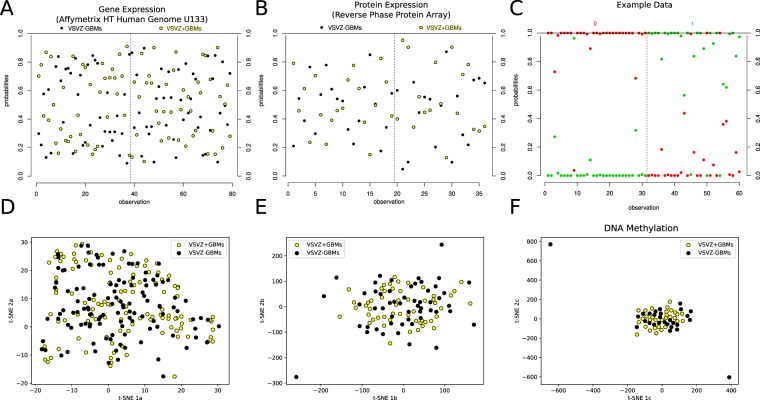
Computational analyses do not distinguish ventricular-subventricular zone-contacting glioblastomas (VSVZ + GBMs) in the TCIA/TCGA samples. Partial least squares followed by logistic regression (PLS-LR) models trained on two-thirds of samples are unable to reliably predict VSVZ + GBMs and VSVZ − GBMs in the testing subsets in gene (Affymetrix HT Human Genome U133) and protein expression datasets from TCGA. A PLS-LR model is selected and its predictions are depicted in (**A**,**B**). Samples (observations) are represented on the x-axis segregated by a vertical dotted line based upon if they are VSVZ + GBMs or VSVZ − GBMs. The model assigns two probabilities (two dots) to each sample of being VSVZ + GBM (yellow) or VSVZ − GBM (black). Therefore, the sum of the probabilities of the two dots per sample is 1. (**C**) is an example of good predictions made by an artificial model of PLS-LR. Here, samples are represented on the x-axis separated by a vertical dotted line into their binary classification of “0” and “1”. The model assigns two probabilities (two dots) to each sample of being 0 or 1. As seen here, the artificial model successfully predicts all the known 0 (red) samples with a high probability of being 0 and a low probability of being 1; and conversely, all the known 1 (green) samples with a high probability of being 1 and a low probability of being 0. (**D**–**F**) represent two-dimensional projection of the high-dimensional gene expression, protein expression, and methylation datasets, respectively, using the t-SNE algorithm (ran with perplexity = 30.0, iterations = 1000). Application of PLS-LR models (linear) or t-SNE (nonlinear multidimension reduction) on molecular datasets does not distinguish VSVZ + GBMs from VSVZ − GBMs.

**Figure 7:**
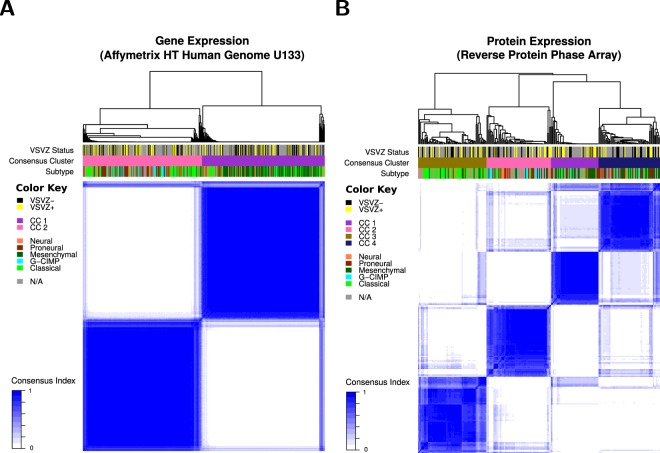
Clustering demonstrates no correlation with ventricular-subventricular zone-contacting glioblastomas (VSVZ + GBMs) or VSVZ − GBMs in the TCIA/TCGA samples. Gene (**A**) and protein (**B**) expression datasets clustered into two and four consensus clusters (CC), respectively, are depicted. The relationship between samples and clusters is depicted with a dendrogram above the image. Underneath, the three bars are used to represent the classification of each sample using a color. Subtype refers to the transcriptional classification. Preponderance of neither VSVZ + GBMs nor VSVZ − GBMs is noted in the two gene or four protein expression clusters.

Therefore, alternative computational approaches were considered. A weighted gene co-expression network analysis^26^ identified 14 modules of co-expressed/coregulated genes (Fig. 5B) but none were enriched in either VSVZ contacting or non-contacting GBMs (Fig. 5C,D, Table S9). A partial least squares followed by logistic regression modeling^27^ using linear combinations of gene and protein expression found no combinations to be predictive of VSVZ + GBM and VSVZ − GBM status (Fig. 6A,B). In addition to these above linear analyses, nonlinear dimensionality reductions were performed using t-SNE^28^, but VSVZ + GBMs remained indistinguishable from VSVZ − GBMs in high-dimensional space based on their gene or protein expression or DNA methylation (Fig. 6D,F). Lastly, unsupervised consensus clustering was performed to identify potential distinct GBM clusters. Although GBMs could be successfully clustered based on gene or protein expression, no cluster was enriched in VSVZ + GBMs or VSVZ − GBMs (Fig. 7). Computational molecular analyses restricted to only G-CIMP negative and IDH wild-type patients were no different from those seen in the entire dataset (Tables S11–S17).

To confirm our approaches, we tested the results of computational analyses such as gene co-expression and consensus clustering on gene expression datasets and found significant correlations with known canonical transcriptional GBM subtype classifications (Fig. 5E,F, Tables S9 and S10). None of these subtypes were predominant in either VSVZ + GBMs or VSVZ − GBMs (*Χ*^2^ *P* = 0.11).

## Discussion

Our survival analysis of VSVZ + GBMs and VSVZ − GBMs was adjusted for universally-accepted predictors of survival in GBM patients, including patient performance status (i.e., KPS), tumor volume, and extent of resection, which are potential confounders of an analysis focused on this region. Lower KPS has been observed in patients with VSVZ + GBMs^5,9,11,16^, likely due to their more central location and larger size which could impair the function of the central neural tracts and basal nuclei. In addition, their larger volumes and deeper location may also decrease the likelihood of gross total resections^9,16,31–33^. Unlike prior efforts^5,6,15,16^, our analysis accounted for these and other molecular predictors and demonstrated that contact with the VSVZ by GBM is an independent, negative prognosticator. These results were consistent between our institutional and the TCIA/TCGA-GBM datasets.

To understand the basis for this clinical phenotype, an initial comprehensive molecular analysis between VSVZ + GBMs and VSVZ − GBMs is presented here. Efforts to identify cellular and/or molecular markers associated with VSVZ + GBMs have been numerous^10–17^. However, these studies are limited by lack of VSVZ − GBM controls^10,17^, small sample sizes^10–13,15,17^, evaluation of limited numbers of molecular markers^11–14,16^, and analyses unadjusted for multiple hypothesis testing^15,16^. In this study, which was designed to overcome these limitations, no statistically robust molecular signature emerged distinguishing VSVZ + GBMs from VSVZ − GBMs with the available sample size.

The distinct survival phenotypes of VSVZ + GBMs and VSVZ − GBMs and the absence of molecular signatures which identify them offer an important guide to future research aimed at understanding the role of VSVZ contact in GBM pathobiology. These results suggest that VSVZ + GBMs and VSVZ − GBMs are not transcriptionally or genomically distinct when considered as bulk samples; therefore, the unique cytoarchitecture and molecular properties of the VSVZ deserve increased attention. Additionally, enrichment for a region-specific phenotype may not be detectable in the bulk tumor samples examined here.

The growth factor-rich environment of the niche may uniquely support and/or enhance the neoplastic potential of GBMs. The normal VSVZ supports cells which are responsive to epidermal growth factor (EGF), platelet-derived growth factor (PDGF), and Ephrins, among other growth factors and chemokines^34,35^. With the exception of EGFR amplification, a greater proportion of receptor tyrosine kinase gene amplifications, specifically *PDGFR-α*, *EPHB3*, and *KIT*, was noted in VSVZ + GBMs, but did not meet our adjusted statistical thresholds (Tables S5 and S12). These results suggest that VSVZ-enriched growth factors could support the aberrant, neoplastic growth signaling of GBM cells through these amplified growth factor receptors. GBM cells are similarly known to be attracted to the niche by VSVZ growth factors. This has been observed radiographically in humans^5,36^ and in mouse xenograft models, which revealed specific mediators including VSVZ derived stromal cell-derived factor 1 (SDF1 or CXCL12)^37^ and neurite growth-promoting factor 1 (NEGF1 or Pleiotrophin)^38^. Interestingly in a mouse model, SDF1 released by the VSVZ mediated resistance to radiation therapy in the GBM cells located in the VSVZ^39^. This may partly explain why 8 of the at least 12 clinical studies conducted to date failed to demonstrate a benefit of incorporating VSVZ radiation in the standard GBM therapy regimen^7,8^.

Upon the arrival of cancer cells in this niche, the VSVZ’s fertile microenvironment, cytoarchitecture (including a well-characterized gap layer in the adult human brain), and extensive contact with the circulating CSF likely make it uniquely permissive for cell migration, including sub-ependymal spread^40–42^ and widespread dissemination. Several observations support the hypothesis that the VSVZ permits spread of glioma cells. First, VSVZ contact is associated with multifocal GBM^5,43–45^. Second, VSVZ + GBMs are associated with distant recurrences post-therapy^43,44,46,47^. The exact VSVZ structural components that allow GBM cells to spread and disseminate once in this region remain unknown. Although multiple factors controlling the rostral migration of young neurons in this niche have been identified in the mouse, it is less clear which of these factors are persistently expressed in adult human brain. Finally, surgical violation of the VSVZ resulting in an entry of tumor cells to the circulating CSF is also associated with widespread dissemination and distant recurrences^31,48,49^.

VSVZ neurogenesis and its other reparative functions decline precipitously with age in humans^50,51^. The niche may therefore be more fertile in younger patients, meaning that GBMs may draw more malignant potential from components of the VSVZ niche, such as the cerebrospinal fluid, in these cases. The age-dependent effect of VSVZ contact by GBMs on patient survival, where younger VSVZ + GBM patients in both datasets had a significantly greater rate of mortality compared to older VSVZ + GBM patients, supports this inference. However, a more detailed examination of the effects of host age will likely be necessary to investigate potential molecular mechanisms driving this clinical finding.

This study reveals contact with the V-SVZ as an independent predictor of outcome without obvious molecular correlates, indicating that VSVZ + GBMs and VSVZ − GBMs may not be intrinsically different on the analysis platforms tested. One hypothesis derived from these findings is that the VSVZ, and the secreted factors enriched within it, may elicit stem-like functional features in otherwise similar tumor cells. Alternatively, the VSVZ may support transcriptionally distinct subsets of GBM cells which are not readily detectable in bulk analyses. Therefore, further aggregate or single-cell analyses coupled with position-specific sampling are warranted. In support of this latter hypothesis, we note that a focused study of sphere cultures derived from subregions of GBM tumors suggested possible enrichment for the mesenchymal subtype in cultures derived from VSVZ-proximal regions in comparison to VSVZ-distant regions of the same tumor^52^. However, additional research on this topic is needed to separate the potential contribution of any resident non-cancerous neural stem cells, which share many features with the mesenchymal signature, to such molecular analyses. Future studies examining the cytoarchitectural structure and growth factor-rich environment of the VSVZ together with its influence on the functional phenotypes of GBM cells (e.g. quiescence, radiation resistance, and metabolic alterations)^53^ are warranted to shed light on the distinct survival profiles of VSVZ + GBMs and VSVZ − GBMs. Finally, single-cell rather than homogenizing methods will be of value when identifying the exact GBM cells influenced by the VSVZ. In addition to single-cell RNA sequencing or lineage barcoding, capturing intracellular signaling and the dynamic modulation of stem-like features by VSVZ growth factors in GBM cells will likely generate valuable information. These investigations will be key components in the search to identify the mechanistic underpinnings of the observed survival phenotype, dissemination, and therapy resistance.

## Conclusion

This comprehensive survival and molecular analysis finds that survival of GBM patients is independently stratified by VSVZ contact of the tumor. Specifically, patients with VSVZ + GBMs have a significantly lower survival. A thorough analysis of the TCGA molecular data did not reveal a molecular signature specific to the VSVZ + GBMs.

## Supplementary information

Supplemental Information

Supplementary Dataset 1

## References

[1] Sanai N (2004). Unique astrocyte ribbon in adult human brain contains neural stem cells but lacks chain migration. Nature.

[2] Eriksson PS (1998). Neurogenesis in the adult human hippocampus. Nature medicine.

[3] Vescovi AL, Galli R, Reynolds BA (2006). Brain tumour stem cells. Nature reviews. Cancer.

[4] Sanai N, Alvarez-Buylla A, Berger MS (2005). Neural stem cells and the origin of gliomas. The New England journal of medicine.

[5] Mistry AM (2017). Decreased survival in glioblastomas is specific to contact with the ventricular-subventricular zone, not subgranular zone or corpus callosum. Journal of neuro-oncology.

[6] Mistry AM (2017). Influence of glioblastoma contact with the lateral ventricle on survival: a meta-analysis. Journal of neuro-oncology.

[7] Nourallah B, Digpal R, Jena R, Watts C (2017). Irradiating the Subventricular Zone in Glioblastoma Patients: Is there a Case for a Clinical Trial?. Clinical oncology.

[8] Foro Arnalot, P. *et al*. Influence of incidental radiation dose in the subventricular zone on survival in patients with glioblastoma multiforme treated with surgery, radiotherapy, and temozolomide. *Clinical & translational oncology: official publication of the Federation of Spanish Oncology Societies and of the National Cancer Institute of Mexico*, 10.1007/s12094-017-1659-5 (2017). 10.1007/s12094-017-1659-5 28389881

[9] Mistry, A. M. Clinical correlates of subventricular zone-contacting glioblastomas: a meta-analysis. *Journal of neurosurgical sciences*, 10.23736/S0390-5616.17.04274-6 (2017). 10.23736/S0390-5616.17.04274-6 29205011

[10] Haskins WE (2013). Molecular Characteristics in MRI-Classified Group 1 Glioblastoma Multiforme. Frontiers in oncology.

[11] Kappadakunnel M (2010). Stem cell associated gene expression in glioblastoma multiforme: relationship to survival and the subventricular zone. Journal of neuro-oncology.

[12] Tomita T, Akimoto J, Haraoka J, Kudo M (2014). Clinicopathological significance of expression of nestin, a neural stem/progenitor cell marker, in human glioma tissue. Brain tumor pathology.

[13] Han S, Li X, Qiu B, Jiang T, Wu A (2015). Can lateral ventricle contact predict the ontogeny and prognosis of glioblastoma?. Journal of neuro-oncology.

[14] Pina Batista KM (2015). Prognostic significance of the markers IDH1 and YKL40 related to the subventricular zone. Folia neuropathologica/Association of Polish Neuropathologists and Medical Research Centre, Polish Academy of Sciences.

[15] Gollapalli K (2017). Subventricular zone involvement in Glioblastoma - A proteomic evaluation and clinicoradiological correlation. Scientific reports.

[16] Jungk C (2016). Spatial transcriptome analysis reveals Notch pathway-associated prognostic markers in IDH1 wild-type glioblastoma involving the subventricular zone. BMC medicine.

[17] Lin CA, Rhodes CT, Lin C, Phillips JJ, Berger MS (2017). Comparative analyses identify molecular signature of MRI-classified SVZ-associated glioblastoma. Cell cycle (Georgetown, Tex.).

[18] Stupp R (2005). Radiotherapy plus concomitant and adjuvant temozolomide for glioblastoma. The New England journal of medicine.

[19] Clark K (2013). The Cancer Imaging Archive (TCIA): maintaining and operating a public information repository. Journal of digital imaging.

[20] Ceccarelli M (2016). Molecular Profiling Reveals Biologically Discrete Subsets and Pathways of Progression in Diffuse Glioma. Cell.

[21] Medical Learning Network. Global Surgery Booklet. Centers for Medicare & Medicaid Services. ICN 907166. 1–17 (August 2017).

[22] Cerami E (2012). The cBio cancer genomics portal: an open platform for exploring multidimensional cancer genomics data. Cancer discovery.

[23] Gao J (2013). Integrative analysis of complex cancer genomics and clinical profiles using the cBioPortal. Science signaling.

[24] Benjamini Y, Hochberg Y (1995). Controlling the False Discovery Rate: A Practical and Powerful Approach to Multiple Testing. J R Stat Soc Ser B.

[25] Thomas L, Reyes EM (2014). Tutorial: Survival Estimation for Cox Regression Models with Time-Varying Coefficients Using SAS and R. 2014.

[26] Langfelder P, Horvath S (2008). WGCNA: an R package for weighted correlation network analysis. BMC bioinformatics.

[27] Slawski M, Daumer M, Boulesteix AL (2008). CMA: a comprehensive Bioconductor package for supervised classification with high dimensional data. BMC bioinformatics.

[28] van der Maaten L, Hinton G (2008). Visualizing data using t-SNE. The Journal of Machine Learning Research.

[29] Wilkerson MD, Hayes DN (2010). ConsensusClusterPlus: a class discovery tool with confidence assessments and item tracking. Bioinformatics.

[30] Bourgon R, Gentleman R, Huber W (2010). Independent filtering increases detection power for high-throughput experiments. Proceedings of the National Academy of Sciences of the United States of America.

[31] Adeberg S (2014). Glioblastoma recurrence patterns after radiation therapy with regard to the subventricular zone. International journal of radiation oncology, biology, physics.

[32] Jafri NF, Clarke JL, Weinberg V, Barani IJ, Cha S (2013). Relationship of glioblastoma multiforme to the subventricular zone is associated with survival. Neuro-oncology.

[33] Ho J (2013). Chemoirradiation for glioblastoma multiforme: the national cancer institute experience. PloS one.

[34] Tong CK, Alvarez-Buylla A (2014). SnapShot: adult neurogenesis in the V-SVZ. Neuron.

[35] Sinnaeve, J., Mobley, B. C. & Ihrie, R. A. Space Invaders: Brain Tumor Exploitation of the Stem Cell Niche. *The American journal of pathology*, 10.1016/j.ajpath.2017.08.029 (2017). 10.1016/j.ajpath.2017.08.029 PMC5745521 29024634

[36] Parsa AT (2005). Prognostic significance of intracranial dissemination of glioblastoma multiforme in adults. Journal of neurosurgery.

[37] Goffart N (2015). Adult mouse subventricular zones stimulate glioblastoma stem cells specific invasion through CXCL12/CXCR4 signaling. Neuro-oncology.

[38] Qin EY (2017). Neural Precursor-Derived Pleiotrophin Mediates Subventricular Zone Invasion by Glioma. Cell.

[39] Goffart N (2017). CXCL12 mediates glioblastoma resistance to radiotherapy in the subventricular zone. Neuro-oncology.

[40] Gilbertson RJ, Rich JN (2007). Making a tumour’s bed: glioblastoma stem cells and the vascular niche. Nature reviews. Cancer.

[41] Iacoangeli M (2012). Endoscopy-verified occult subependymal dissemination of glioblastoma and brain metastasis undetected by MRI: prognostic significance. OncoTargets and therapy.

[42] Hayashi Y (2010). Implication of 5-aminolevulinic acid fluorescence of the ventricular wall for postoperative communicating hydrocephalus associated with cerebrospinal fluid dissemination in patients with glioblastoma multiforme: a report of 7 cases. Journal of neurosurgery.

[43] Liang TH (2016). Adverse prognosis and distinct progression patterns after concurrent chemoradiotherapy for glioblastoma with synchronous subventricular zone and corpus callosum invasion. Radiotherapy and oncology: journal of the European Society for Therapeutic Radiology and Oncology.

[44] Adeberg S (2014). A comparison of long-term survivors and short-term survivors with glioblastoma, subventricular zone involvement: a predictive factor for survival?. Radiation oncology (London, England).

[45] Lim DA (2007). Relationship of glioblastoma multiforme to neural stem cell regions predicts invasive and multifocal tumor phenotype. Neuro-oncology.

[46] Nestler U (2015). Anatomic features of glioblastoma and their potential impact on survival. Acta neurochirurgica.

[47] Sonoda Y (2014). The association of subventricular zone involvement at recurrence with survival after repeat surgery in patients with recurrent glioblastoma. Neurologia medico-chirurgica.

[48] Adeberg S (2016). Is a modification of the radiotherapeutic target volume necessary after resection of glioblastomas with opening of the ventricles?. Journal of neuro-oncology.

[49] Roelz R (2015). Surgical Ventricular Entry is a Key Risk Factor for Leptomeningeal Metastasis of High Grade Gliomas. Scientific reports.

[50] Conover JC, Shook BA (2011). Aging of the Subventricular Zone Neural Stem Cell Niche. Aging and disease.

[51] Capilla-Gonzalez V, Herranz-Perez V, Garcia-Verdugo JM (2015). The aged brain: genesis and fate of residual progenitor cells in the subventricular zone. Frontiers in cellular neuroscience.

[52] Piccirillo SG (2015). Contributions to drug resistance in glioblastoma derived from malignant cells in the sub-ependymal zone. Cancer research.

[53] Lathia JD, Mack SC, Mulkearns-Hubert EE, Valentim CL, Rich JN (2015). Cancer stem cells in glioblastoma. Genes & development.

