# Supplementary material for: Ventricular-Subventricular Zone Contact by Glioblastoma is Not Associated with Molecular Signatures in Bulk Tumor Data
Source: Sci Rep. 2019 Feb 12;9:1842. doi: 10.1038/s41598-018-37734-w (PMC6372607; doi:10.1038/s41598-018-37734-w)
Supplement: Supplementary file 1 — Supplemental Information [file 41598_2018_37734_MOESM1_ESM.pdf]

SUPPLEMENTARY INFORMATION

Manuscript Title: Ventricular-Subventricular Zone Contact by Glioblastoma is Not Associated with Molecular Signatures in Bulk Tumor Data

Authors: Akshitkumar M. Mistry, MD, David J. Wooten, BS, L. Taylor Davis, MD, Bret C. Mobley, MD, Vito Quaranta, MD, Rebecca A. Ihrie, PhD.

TABLES (in Supplemental Tables.xlsx)

Table S1. Demographic, clinical, and molecular information of 254 glioblastoma patients in The Cancer Imaging Archive and The Cancer Genome Atlas.

Table S2. Molecular datasets from The Cancer Genome Atlas used together with the number of samples and features analyzed in each dataset.

Table S3. Univariate Cox overall survival analyses of VUMC and TCIA/TCGA-GBM datasets.

Table S4. List of gene mutations in ventricular-subventricular zone-contacting GBMs (VSVZ+GBMs) and VSVZ-GBMs.

Table S5. List of copy number variations in ventricular-subventricular zone-contacting GBMs (VSVZ+GBMs) and VSVZ-GBMs.

Table S6. List of genes whose expression varied between ventricular-subventricular zone-contacting GBMs (VSVZ+GBMs) and VSVZ-GBMs.

Table S7. List of proteins whose expression by RPPA varied between ventricular-subventricular zone-contacting GBMs (VSVZ+GBMs) and VSVZ-GBMs.

Table S8. List of methylation sites beta-values differed between ventricular-subventricular zone-contacting GBMs (VSVZ+GBMs) and VSVZ-GBMs.

Table S9. Comparisons of sets of co-expressed genes obtained through a weighted gene co-expression network analysis in ventricular-subventricular zone-contacting glioblastomas (VSVZ+GBMs) and VSVZ-GBMs as well as in GBMs classified by their canonical transcriptional signature.

Table S10. Overlap of clusters obtained through unsupervised consensus clustering with glioblastomas classified by their ventricular-subventricular zone contact (VSVZ+GBM vs. VSVZ-GBMs) and known canonical transcriptional subtypes.

Table S11. List of gene mutations in ventricular-subventricular zone-contacting GBMs (VSVZ+GBMs) and VSVZ-GBMs that are G-CIMP negative and IDH wild-type.

Table S12. List of copy number variations in ventricular-subventricular zone-contacting GBMs (VSVZ+GBMs) and VSVZ-GBMs that are G-CIMP negative and IDH wild-type.

Table S13. List of genes whose expression varied between ventricular-subventricular zone-contacting GBMs (VSVZ+GBMs) and VSVZ-GBMs that are G-CIMP negative and IDH wild-type.

Table S14. List of proteins whose expression by RPPA varied between ventricular-subventricular zone-contacting GBMs (VSVZ+GBMs) and VSVZ-GBMs that are G-CIMP negative and IDH wild-type.

Table S15. List of methylation sites beta-values differed between ventricular-subventricular zone-contacting GBMs (VSVZ+GBMs) and VSVZ-GBMs that are G-CIMP negative and IDH wild-type.

Table S16. Comparisons of sets of co-expressed genes obtained through a weighted gene co-expression network analysis in ventricular-subventricular zone-contacting glioblastomas (VSVZ+GBMs) and VSVZ-GBMs that are G-CIMP negative and IDH wild-type as well as in GBMs classified by their canonical transcriptional signature.

Table S17. Overlap of clusters obtained through unsupervised consensus clustering with glioblastomas classified by their ventricular-subventricular zone contact (VSVZ+GBM vs. VSVZ-GBMs) that are G-CIMP negative and IDH wild-type and known canonical transcriptional subtypes.

FIGURE LEGENDS

Figure S1. Gene expression does not identify ventricular-subventricular zone-contacting glioblastomas (VSVZ+GBMs) in the TCIA/TCGA samples restricted to only G-CIMP negative and IDH wild-type GBMs. (A) Volcano plot of differential gene expression between VSVZ+GBMs and VSVZ-GBMs. Log10 of the unadjusted p-value of gene (dots) is plotted against fold change (the log2 of the ratio of mean gene expression in VSVZ+GBMs and VSVZ-GBMs). Dotted black line represents unadjusted (P=0.05) and red line represents adjusted (P=4.15 x 10-6) threshold of significance. (B) Dendrogram and the results of its dynamic cuts obtained from weighted gene co-expression network analysis of the Affymetrix HT Human Genome U133 gene expression dataset from TCGA. Fourteen modules of coexpressed and coregulated genes defined by the dynamic tree cuts are represented with blocks of color. Module eigengene (expression variation of genes within the module; i.e, high module eigengene expression denotes high expression of genes within that module) of the cyan (C) and tan (D) modules, the two examples depicted, was assessed in each sample and compared between VSVZ+GBMs and VSVZ-GBMs. There is no significant difference in cyan and tan module eigengenes between VSVZ-GBMs and VSVZ+GBMs; however, a significant difference in cyan and tan module eigengenes based on the transcriptional subtype was noted (D and E).

Figure S2. Computational analyses do not distinguish ventricular-subventricular zone-contacting glioblastomas (VSVZ+GBMs) in the TCIA/TCGA samples restricted to only G-CIMP negative and IDH wild-type GBMs. Partial least squares followed by logistic regression (PLS-LR) models trained on two-thirds of samples are unable to reliably predict VSVZ+GBMs and VSVZ-GBMs in the testing subsets in gene (Affymetrix HT Human Genome U133) and protein expression datasets from TCGA. A PLS-LR model is selected and its predictions are depicted in A and B. Samples (observations) are represented on the x-axis segregated by a vertical dotted line based upon if they are VSVZ+GBMs or VSVZ-GBMs. The model assigns two probabilities (two dots) to each sample of being VSVZ+GBM (yellow) or VSVZ-GBM (black). Therefore, the sum of the probabilities of the two dots per sample is 1. C is an example of good predictions made by an artificial model of PLS-LR. Here, samples are represented on the x-axis separated by a vertical dotted line into their binary classification of “0” and “1”. The model assigns two probabilities (two dots) to each sample of being 0 or 1. As seen here, the artificial model successfully predicts all the known 0 (red) samples with a high probability of being 0 and a low probability of being 1; and conversely, all the known 1 (green) samples with a high probability of being 1 and a low probability of being 0. D, E, and F represent two-dimensional projection of the high-dimensional gene expression, protein expression, and methylation datasets, respectively, using the t-SNE algorithm (ran with perplexity=30.0, iterations=1000). Application of PLS-LR models (linear) or t-SNE (nonlinear multidimension reduction) on molecular datasets does not distinguish VSVZ+GBMs from VSVZ-GBMs.

SUPPLEMENTAL METHODS

Computational Analyses of Molecular Datasets

In addition to single gene expression analysis, a weighted gene co-expression network analysis (WGCNA) was performed using its R package (version 1.49),[6] where profile of each gene co-expression module is represented by an eigengene, which is defined as the first principal component of genes within that module. Weak or spurious correlations in the WGCNA analyses were penalized by powers of 7 to 12 such that the resulting networks had scale-free topology (data not shown, available at request). WGCNA modules were computed using the dynamic tree cut algorithm instead of cutting the tree branches at a constant height due to its superior performance.[7] Module eigengene analysis was performed using standard methods.[6]

To detect linear combinations of gene methylations, gene expressions, and protein expressions that could be predictive of VSVZ+GBMs and VSVZ-GBMs, partial least squares followed by logistic regression (PLS-LR)—a technique highly suited for high-dimensional genomic data[8]—was conducted using the Classification for MicroArrays (CMA version 1.34.0) R package.[9] Monte-Carlo cross validation was used to develop training and testing datasets to avoid artificially overfitting the PLS-LR classifier with two-thirds of the samples being retained for the training dataset and with 100 iterations.

To reveal whether VSVZ+GBMs and VSVZ-GBMs segregate in high-dimensional space, nonlinear dimensionality reduction of the multi-dimensional gene methylation, gene expression, and protein expression datasets was performed using t-SNE.[10] t-SNE embedding was calculated using the Barnes-Hut implementation, available from its author Laurens van der Maaten (https://github.com/lvdmaaten/bhtsne). Linear dimensionality reduction was performed first using principal component analysis; then, nonlinear dimensionality reduction was performed using t-SNE on the top 30 latent principal components.

To reveal whether gene and protein expression clustered based on VSVZ contact in GBMs, clustering was performed in these datasets using the ConsensusClusterPlus (v1.40.0)[11] R package. Consensus indices were calculated using *k*-means clustering and Pearson correlation, and final consensus clusters were assembled by hierarchical clustering (average linkage). The cumulative distribution function (CDF) plots aided in finding a stable number of clusters. Empirically, the lowest number of clusters, where the CDF plot was the most stable through all the consensus indices, was chosen for analysis (these were 2 and 4 for gene and protein expression, respectively). CDF plots are available upon request. Consensus clusters were compared with VSVZ status of GBMs using the chi-square (X^2) test of independence.

Except for PLS-LR (used for binary classifications) and nonlinear dimensionality reduction with t-SNE, all other computational methods employed were tested to see whether their results significantly correlated with the known transcriptional GBM subtype classification assigned to each of the samples in the TCIA/TCGA-GBM cohort (neural, proneural, classic, mesenchymal, and G-CIMP).[12] Samples without a designated classification (22 total) were excluded from statistical testing. The R and Python codes used to conduct these analyses are available upon request.

Method References

1 White, I. R., Royston, P. & Wood, A. M. Multiple imputation using chained equations: Issues and guidance for practice. *Statistics in medicine* **30**, 377-399, doi:10.1002/sim.4067 (2011).

2 Bartlett, J. W., Seaman, S. R., White, I. R., Carpenter, J. R. & Alzheimer's Disease Neuroimaging, I. Multiple imputation of covariates by fully conditional specification: Accommodating the substantive model. *Statistical methods in medical research* **24**, 462-487, doi:10.1177/0962280214521348 (2015).

3 Bodner, T. E. What Improves with Increased Missing Data Imputations? *Structural Equation Modeling: A Multidisciplinary Journal* **15**, 651-675, doi:10.1080/10705510802339072 (2008).

4 Allison, P. D. *Missing Data*. (SAGE Publications, 2002).

5 Johnson, D. R. & Young, R. Toward Best Practices in Analyzing Datasets with Missing Data: Comparisons and Recommendations. *Journal of Marriage and Family* **73**, 926-945, doi:10.1111/j.1741-3737.2011.00861.x (2011).

6 Langfelder, P. & Horvath, S. WGCNA: an R package for weighted correlation network analysis. *BMC bioinformatics* **9**, 559, doi:10.1186/1471-2105-9-559 (2008).

7 Langfelder, P., Zhang, B. & Horvath, S. Defining clusters from a hierarchical cluster tree: the Dynamic Tree Cut package for R. *Bioinformatics* **24**, 719-720, doi:10.1093/bioinformatics/btm563 (2008).

8 Boulesteix, A. L. & Strimmer, K. Partial least squares: a versatile tool for the analysis of high-dimensional genomic data. *Briefings in bioinformatics* **8**, 32-44, doi:10.1093/bib/bbl016 (2007).

9 Slawski, M., Daumer, M. & Boulesteix, A. L. CMA: a comprehensive Bioconductor package for supervised classification with high dimensional data. *BMC bioinformatics* **9**, 439, doi:10.1186/1471-2105-9-439 (2008).

10 van der Maaten, L. & Hinton, G. Visualizing data using t-SNE. *The Journal of Machine Learning Research* **9**, 85 (2008).

11 Wilkerson, M. D. & Hayes, D. N. ConsensusClusterPlus: a class discovery tool with confidence assessments and item tracking. *Bioinformatics* **26**, 1572-1573, doi:10.1093/bioinformatics/btq170 (2010).

12 Ceccarelli, M. *et al.* Molecular Profiling Reveals Biologically Discrete Subsets and Pathways of Progression in Diffuse Glioma. *Cell* **164**, 550-563, doi:10.1016/j.cell.2015.12.028 (2016).

Figure S1

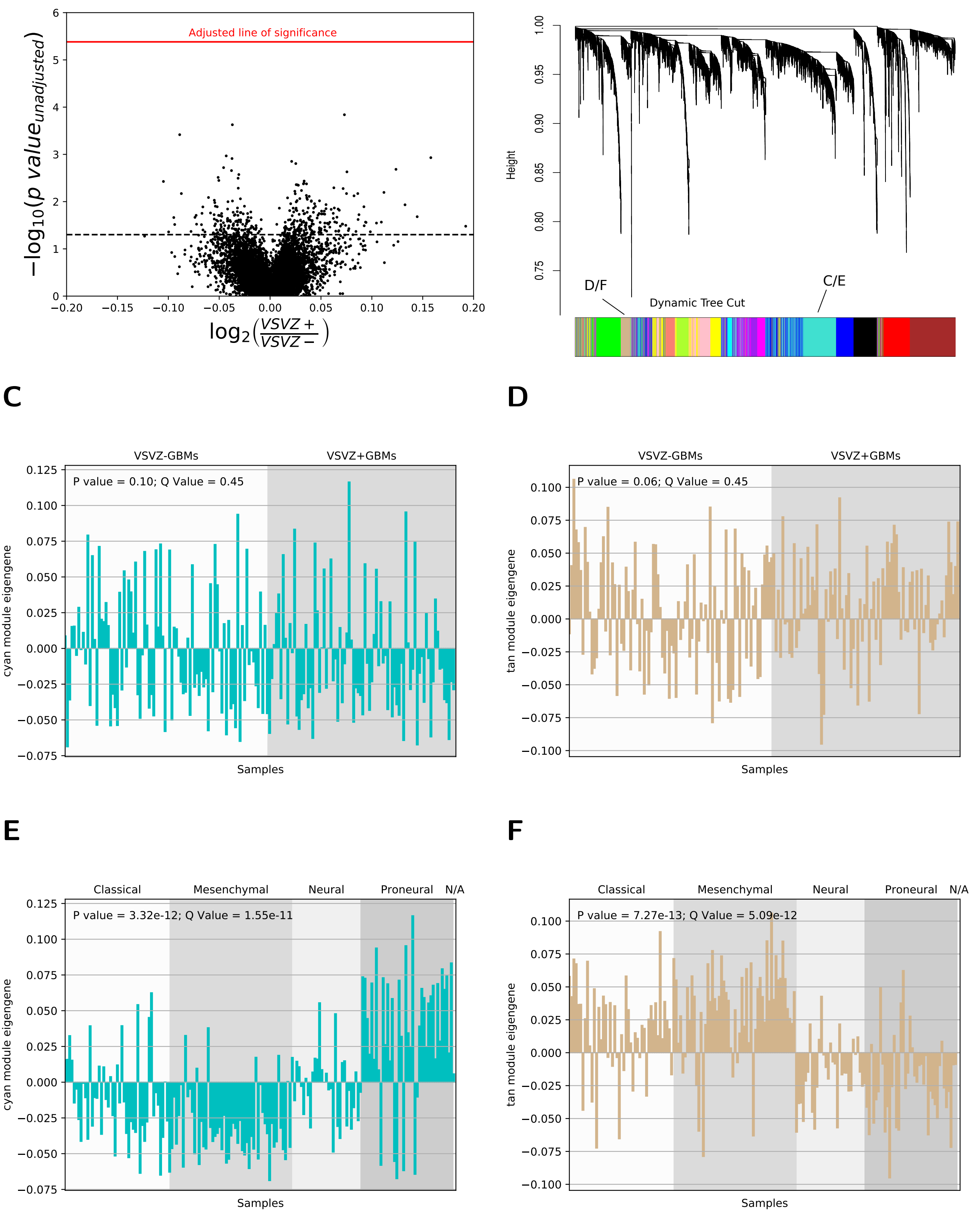

A
Adjusted line of significance
$-\log_{10}(p\ value_{unadjusted})$
$\log_2\left(\frac{VSVZ+}{VSVZ-}\right)$
B
Height
D/F
C/E
Dynamic Tree Cut
C
VSVZ-GBMs
VSVZ+GBMs
P value = 0.10; Q Value = 0.45
cyan module eigengene
Samples
D
VSVZ-GBMs
VSVZ+GBMs
P value = 0.06; Q Value = 0.45
tan module eigengene
Samples
E
Classical
Mesenchymal
Neural
Proneural
N/A
P value = 3.32e-12; Q Value = 1.55e-11
cyan module eigengene
Samples
F
Classical
Mesenchymal
Neural
Proneural
N/A
P value = 7.27e-13; Q Value = 5.09e-12
tan module eigengene
Samples

Figure S2

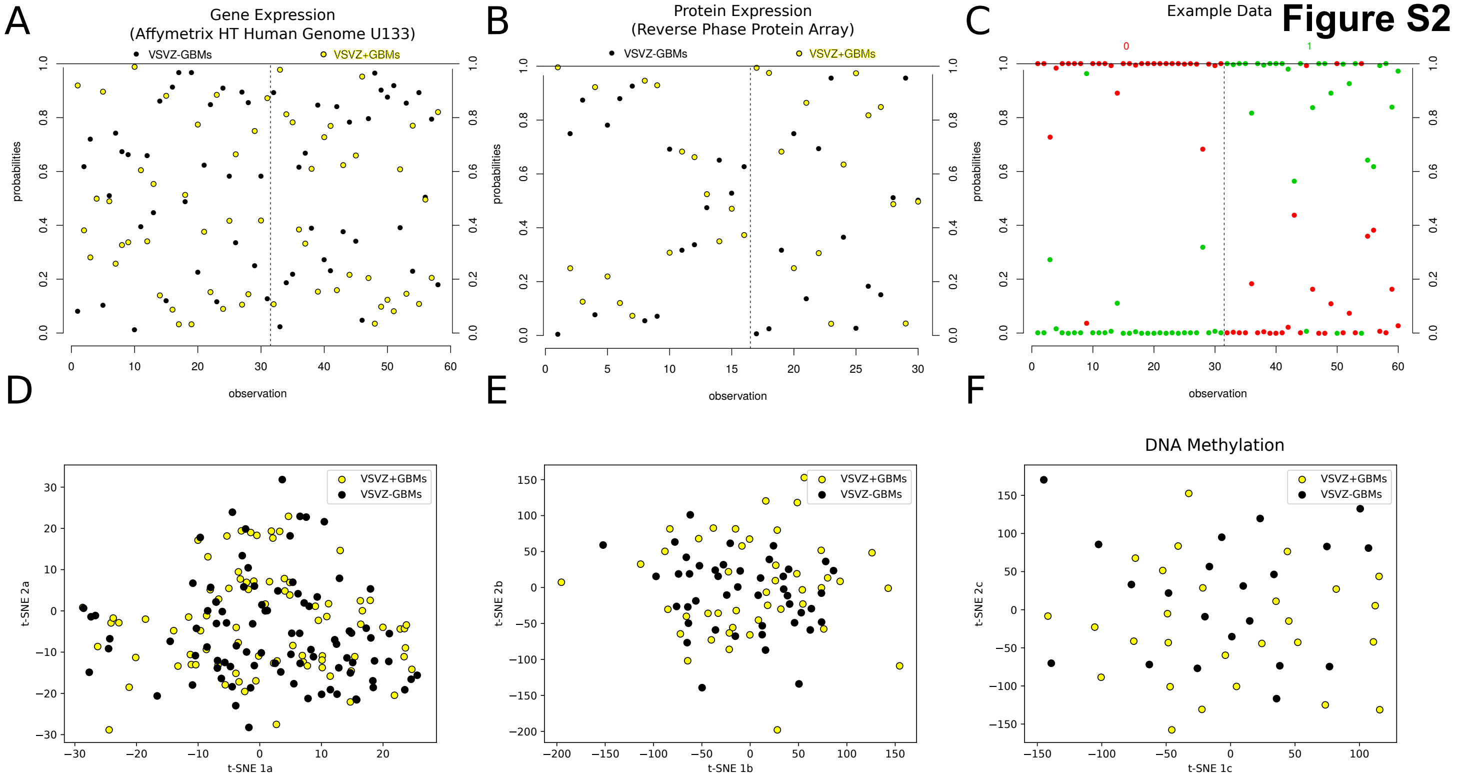

A
Gene Expression
(Affymetrix HT Human Genome U133)
VSVZ-GBMs
VSVZ+GBMs
probabilities
observation
B
Protein Expression
(Reverse Phase Protein Array)
VSVZ-GBMs
VSVZ+GBMs
probabilities
observation
C
Example Data
0
1
probabilities
observation
D
VSVZ+GBMs
VSVZ-GBMs
t-SNE 2a
t-SNE 1a
E
VSVZ+GBMs
VSVZ-GBMs
t-SNE 2b
t-SNE 1b
F
DNA Methylation
VSVZ+GBMs
VSVZ-GBMs
t-SNE 2c
t-SNE 1c
